# Blood flow‐induced angiocrine signals promote organ growth and regeneration

**DOI:** 10.1002/bies.202400207

**Published:** 2024-11-11

**Authors:** Paula Follert, Linda Große‐Segerath, Eckhard Lammert

**Affiliations:** ^1^ Heinrich Heine University Düsseldorf, Faculty of Mathematics and Natural Sciences Institute of Metabolic Physiology Düsseldorf Germany; ^2^ German Diabetes Center (DDZ) Leibniz Center for Diabetes Research at Heinrich Heine University Düsseldorf Düsseldorf Germany; ^3^ German Center for Diabetes Research (DZD e.V.) Neuherberg Germany

**Keywords:** angiocrine signals, blood flow, liver, Myeloid‐derived growth factor (MYDGF), organ growth, regeneration

## Abstract

Recently, we identified myeloid‐derived growth factor (MYDGF) as a blood flow‐induced angiocrine signal that promotes human and mouse hepatocyte proliferation and survival. Here, we review literature reporting changes in blood flow after partial organ resection in the liver, lung, and kidney, and we describe the angiocrine signals released by endothelial cells (ECs) upon blood flow alterations in these organs. While hepatocyte growth factor (HGF) and MYDGF are important angiocrine signals for liver regeneration, by now, angiocrine signals have also been reported to stimulate hyperplasia and/or hypertrophy during the regeneration of lungs and kidneys. In addition, angiocrine signals play a critical role in tumor growth. Understanding the mechano‐elastic properties and flow‐mediated alterations in the organ‐specific microvasculature is crucial for therapeutic approaches to maintain organ health and initiate organ renewal.

## Graphical abstract

Partial hepatectomy leads to hemodynamic changes in the remaining liver that stretch hepatic endothelial cells and trigger the release of angiocrine signals to promote liver regeneration. This review examines whether this mechanism might also explain compensatory growth after partial loss of the lung and kidney.

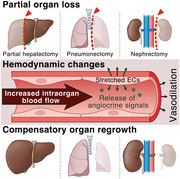

## INTRODUCTION

Normal blood flow is essential for the delivery of nutrients and oxygen to any given organ and is regulated by multiple endogenous mechanisms to adjust blood flow to the physiological needs of the organ. Severe changes in blood flow can be observed after partial organ resection, leading to extreme hemodynamic changes (that is differences in blood flow‐induced shear forces and blood pressure) in the remaining organ tissue. Specifically, when parts of an organ are removed, the blood that previously flowed through the entire organ is forced to pass the remaining smaller part of the organ until the stage when the organ with its entire vasculature is restored.^[^ ^1^, ^2^, ^3^ ^]^ In many cases, tissues and organs experiencing these hemodynamic changes regrow or at least show some kind of compensatory growth. Recently, the effects of organ resection on blood flow and blood vessels have been of particular interest to understand how complex adaptive responses of the microvasculature lead to full regeneration of some organs and tissues.^[^ ^4^, ^5^ ^]^

Endothelial cells (ECs) are thought to be a key cell type controlling organ regeneration. First, ECs line the lumen of each blood vessel and their apical cell surface is orientated toward flowing blood. In a slim human individual, the endothelial surface is—to the largest extent—formed by the EC area of the microvasculature and is estimated to be around 1000 m^2^, which is larger than the area of a handball field.^[^ ^6^ ^]^ By comparison, the human skin only covers an area of around 1.5–2 m^2^,^[^ ^7^ ^]^ which is smaller than the area of a ping‐pong table. Second, ECs express mechanosensory proteins like integrins, ion channels, cell junctional proteins, G‐protein coupled receptors (GPCRs), growth factor receptors as well as multiple cytosolic and nuclear proteins that respond to changes in mechanical forces.^[^ ^8^ ^]^ Finally, the response of ECs to multiple stimuli, including mechanical ones, leads to the release of growth factors, cytokines, and chemokines, referred to as “angiocrine signals”. Notably, these signals can positively or negatively influence the proliferation, differentiation, and survival of organ‐specific cell types during organ growth and regeneration as well as organ‐specific diseases ^[^ ^9^, ^10^, ^11^ ^]^. Due to their physiologic and pathologic relevance, angiocrine signals have now been investigated for more than 20 years.^[^ ^12^, ^13^, ^14^ ^]^ For the regeneration of organs, ECs as cell types that both sense blow flow‐related alterations and respond to these by secretion of angiocrine signals, have recently been of particular interest (Table 1).^[^ ^8^, ^15^, ^16^ ^]^ The release of these angiocrine signals is likely to be mediated by ECs lining capillaries, as these form the largest and closest interface between blood and organ‐specific cell types.^[^ ^14^ ^]^ To our knowledge, the extent to which larger vessels, such as arteries, secrete angiocrine signals and how these affect adult organ growth and regeneration after partial tissue loss has not been well characterized to date.

**TABLE 1:** Angiocrine signals and their function after partial organ loss of liver, lung and kidney.

Organ	Angiocrine factor	Function after tissue loss	Reference
**Liver**	HGF	Promotes liver regeneration after PHx; LSEC‐specific deletion of *Hgf* delays liver regeneration; Deletion of *Id1* results in reduced HGF expression and impairs liver regeneration in mice after PHx; Notch1 activation in ECs reduces HGF release from LSECs	^[^ ^20^, ^40^, ^41^, ^42^, ^53^ ^]^
	Wnt2, Wnt9b	Essential for hepatic zonation and liver regeneration; Wnt2 and Wnt9b get secreted after PHx; EC‐specific deficiency of GATA4 leads to downregulation of Wnt2, resulting in impaired metabolic liver zonation and liver regeneration; Deletion of *Id1* reduces Wnt2 expression and impairs liver regeneration in mice after PHx; Notch1 activation in ECs lowers Wnt2 and Wnt9b release from LSECs	^[^ ^40^, ^48^, ^49^, ^50^, ^53^ ^]^
	MYDGF	Promotes proliferation of primary human hepatocytes; Overexpression of *Mydgf* in the liver improves hepatocyte proliferation after PHx; Knockout of *Mydgf* nearly abolishes hepatocyte proliferation after PHx	^[^ ^55^ ^]^
	IL‐6	Upregulation of HGF after PHx depends on IL‐6 trans‐signaling; *Il6* deficient mice show impaired liver regeneration after PHx; Mice with depletion of IL‐6 receptor (IL‐6R) have a reduced survival rate after PHx	^[^ ^20^, ^123^, ^124^ ^]^
	TNFα	Stimulates priming of hepatocytes and HGF expression after PHx; promitogenic effect of TNF on hepatocytes depends on NFκB activation after PHx	^[^ ^19^, ^20^, ^125^ ^]^
	Ang‐2	Ang‐2 in LSECs is downregulated in early liver regeneration, reducing TGFβ1 production in LSECs and promoting hepatocyte proliferation; In the later phase of liver regeneration, Ang‐2 is upregulated and promotes angiogenesis through the expression of VEGFR2	^[^ ^126^ ^]^
	TGFβ1	Promotes LSECs to secrete IL‐6 in vitro; TGFβ1 expression in LSECs is regulated by Ang‐2 during liver regeneration; Increased TGFβ1 levels inhibit hepatocyte proliferation	^[^ ^1^, ^126^, ^127^ ^]^
	RSPO3, RSPO1	Pericentral LSECs are the main source of *Rspo3*; global deletion of *Rspo3* impairs liver regeneration through impaired Wnt signaling in hepatocytes; recombinant RSPO1 protein improves liver size and regeneration after PHx	^[^ ^128^, ^129^, ^130^ ^]^
	HB‐EGF	HB‐EGF gets secreted by stretched LSECs after PHx to promote liver regeneration; HB‐EGF expression is mediated by YAP activation and nuclear translocation	^[^ ^131^ ^]^
	IGF1	IGF1 promotes liver regeneration via upregulation of HGF and downregulation of TGFβ1 expression; Growth hormone‐IGF1‐IGF1R axis is necessary for liver regeneration after PHx in mice with a liver‐specific IGF‐1R knockout	^[^ ^132^, ^133^, ^134^ ^]^
	BMP	Hepatocyte proliferation is promoted by BMP‐7, while BMP‐2, BMP‐4 and BMP‐9 inhibit hepatocyte proliferation	^[^ ^135^ ^]^
**Lung**	HGF	Recombinant HGF stimulates DNA synthesis in mouse lung epithelial cells after PNX	^[^ ^136^ ^]^
	MMP14	Unmasks cryptic EGFR ligands to stimulate AT2 cell proliferation; MMP14 production after PNX is reduced in mice with an EC‐specific inducible genetic ablation of *Vegfr2* and *Fgfr1*	^[^ ^88^, ^89^ ^]^
	HB‐EGF	Stimulates alveolar epithelial regeneration	^[^ ^88^, ^89^ ^]^
**Kidney**	HGF	Exogenous HGF treatment increases DNA synthesis in tubular epithelial cells after nephrectomy in mice; HGF levels increase in rat kidney ECs after nephrectomy	^[^ ^106^, ^137^ ^]^
	TGFβ	Induces hypertrophy in renal tubular cells	^[^ ^107^ ^]^
	IGF1	*Igf1* is mainly expressed in medullary renal ECs; *Igf1* deficient mice have a smaller kidney size under basal conditions compared to control mice; however, compensatory regrowth of the kidney after nephrectomy is not impaired in these mice	^[^ ^138^, ^139^, ^140^ ^]^
	GH	Compensatory renal growth in adult rats depends on GH	^[^ ^141^ ^]^

Abbreviations: Ang‐2, Angiopoietin‐2; BMP, Bone morphogenetic protein; ECs, endothelial cells; EGFR, epidermal growth factor receptor; FGFR, Fibroblast Growth Factor receptor; GH, Growth hormone; HB‐EGF, Heparin‐binding epidermal growth factor; HGF, hepatocyte growth factor; IGF1, Insulin‐like growth factor 1; IGF1R, insulin‐like growth factor 1 receptor; IL‐6, Interleukin 6; IL‐6R, interleukin‐6 receptor; LSECs, liver sinusoidal endothelial cells; MMP14, Matrix metalloproteinase 14; MYDGF, myeloid‐derived growth factor; PHx, partial hepatectomy; PNX, pneumonectomy; RSPO3, Rspondin 3; RSPO1, Rspondin 1; TNFα, Tumor necrosis factor alpha; TGFβ1, Transforming‐growth factor beta 1; VEGFR, vascular endothelial growth factor receptor; YAP, Yes‐associated protein.

In this review, we focus on the link between hemodynamic alterations and the release of angiocrine signals reported to initiate organ growth and regeneration after partial organ resection. We have chosen three different organs, that is, the liver, the lung, and the kidney, which show different abilities to regenerate. By understanding how hemodynamic changes influence the secretion profile of ECs, we hope to gain a better understanding of the body's adaptive (versus maladaptive) mechanisms of organ regeneration and also to improve regenerative medicine through targeted interventions.

## LIVER REGENERATION IS INITIATED BY HEMODYNAMIC CHANGES AND ANGIOCRINE SIGNALS AFTER LIVER RESECTION

The liver has a remarkable capacity to rapidly regenerate after surgical liver resection. In rodents, the liver fully regenerates within 5–7 days after the removal of two‐thirds of the liver (which is called partial hepatectomy, PHx).^[^ ^17^ ^]^. But also in humans, the liver regrows within 2 weeks after surgically removing more than half of the liver.^[^ ^17^ ^]^

**FIGURE 1:**
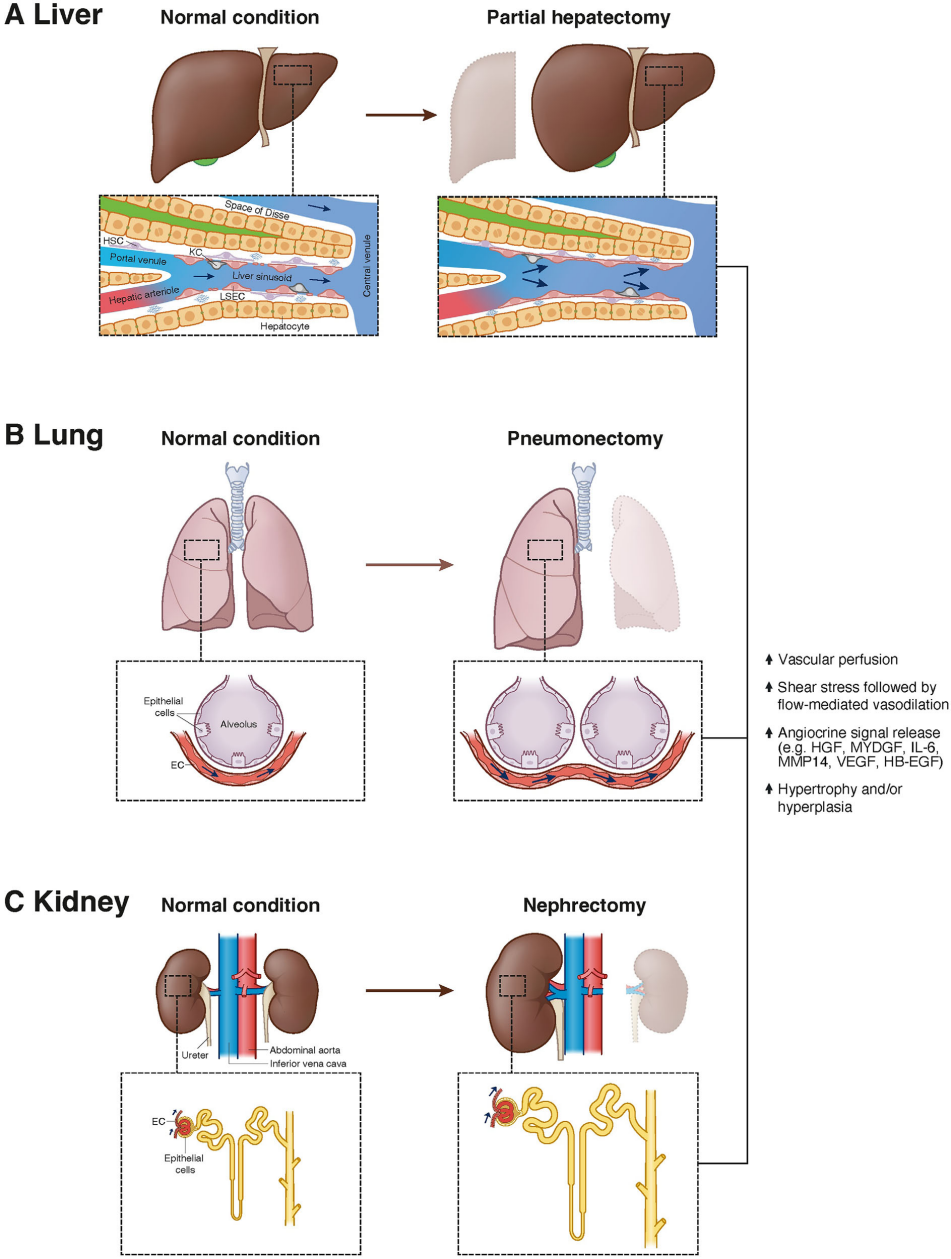
Blood flow‐induced angiocrine signals governing organ growth after partial organ resection. (A) Under normal conditions, the blood in the liver flows from the portal venule and hepatic arteriole through the liver sinusoids to the central venule or vein. The apical surface of the LSECs faces the bloodstream, and the LSECs are separated from hepatocytes by the space of Disse. After a PHx, the sinusoids are vasodilated and, therefore, the LSECs are mechanically stretched. This stimulation triggers the release of several angiocrine signals, that is HGF, MYDGF and IL‐6. Angiocrine signals promote hypertrophy and hyperplasia of the liver. A similar mechanism might take place in lung and kidney. (B) In the lung, ECs also face the bloodstream with their apical cell surface. After PNX, mechanical forces (including those induced by the altered blood flow) cause stretching of alveolar epithelial cells and ECs. Angiocrine signals, that is MMP14 and HB‐EGF, are released to contribute to the growth of the lung. (C) In the kidney, ECs also face the bloodstream with their apical surface. After nephrectomy, hemodynamic changes occur in the remaining kidney, which may trigger the release of angiocrine signals. For the kidney, HGF has been identified as an angiocrine signal. EC, endothelial cells; HB‐EGF, heparin‐binding epidermal growth factor; HGF, hepatocyte growth factor; IL‐6, interleukin‐6; MMP14, matrix metalloprotease 14; MYDGF, myeloid‐derived growth factor; LSEC, liver sinusoidal endothelial cells; PHx, partial hepatectomy;PNX, pneumonectomy.

Liver regeneration is thought to be initiated by a complex interplay of hemodynamic changes, cellular signaling events, and structural adaptions, especially within and around the hepatic blood vessels (Figure 1A). The intact human liver receives approximately 1.25 L of blood every minute.^[^ ^18^ ^]^ Even when two‐thirds of the liver is dissected, around the same volume of blood continues to flow at the same rate per minute through the remaining one‐third of the liver in order to get back to the heart.^[^ ^19^ ^]^ Therefore, the vasculature of the remaining liver tissue must accommodate a greater blood volume flowing through a substantially smaller vascular bed.^[^ ^5^, ^20^ ^]^ These hemodynamic changes are most relevant for the small blood vessels of the hepatic microvasculature, that is, the liver sinusoids, which harbor about 60% of the total blood in the liver. The latter are lined by fenestrated liver sinusoidal endothelial cells (LSECs) and their adjacent pericytes (or hepatic stellate cells) and closely interact with the hepatic parenchymal cells, that is, hepatocytes.^[^ ^9^, ^20^, ^21^ ^]^

On a cellular level, hepatocyte proliferation is one of the initial events in liver regeneration after resection of a large part of the liver and peaks within the first 2–3 days,^[^ ^22^ ^]^ which is followed by the proliferation of LSECs with some delay.^[^ ^23^ ^]^ Initiation of hepatocyte proliferation is thought to be triggered by ECs that release angiocrine growth factors for the hepatocytes.^[^ ^5^, ^20^, ^24^ ^]^ After initiating hepatocyte proliferation, ECs turn angiogenic and start to proliferate. This happens within the first 4–7 days after liver resection, that is when they experience vascular endothelial growth factor‐A (VEGF‐A) secreted by proliferating hepatocytes, activation of angiopoietin‐2 (Ang2) and vascular endothelial growth factor receptor‐2 (VEGFR2) signaling.^[^ ^23^ ^]^ Taken together, the proliferation of hepatocytes (and thus the restoration of the liver parenchyma) occurs first and is triggered by angiocrine ECs, followed by the proliferation of angiogenic ECs (and other non‐parenchymal cell types) to finally restore the original size of the hepatic vasculature.

In healthy liver tissue, only a small number of hepatocytes proliferate to maintain the hepatocyte mass, but after liver resection following two‐thirds PHx, there is a massive proliferation of hepatocytes.^[^ ^19^ ^]^ In each liver lobule, hepatocytes are organized in three different zones—zone 1 near a portal venule, zone 2 midlobular, and zone 3 near a central venule (also called central vein). Interestingly, midlobular hepatocytes contribute the most to liver regrowth within the first 3 days after PHx.^[^ ^25^, ^26^ ^]^ In addition to the proliferation of hepatocytes (called hepatic hyperplasia^[^ ^27^ ^]^), hepatic hypertrophy (that is, the increase in size of hepatocytes) happens after PHx, with the largest increase in cell size observed in midlobular hepatocytes.^[^ ^28^ ^]^ However, hepatocytes throughout the entire liver contribute to liver regrowth, and proliferation is not restricted to stem cell‐like populations.^[^ ^29^ ^]^

It is noteworthy that the amount of resected liver mass triggers different growth stimuli. While a two‐thirds PHx rapidly leads to hypertrophy followed by hyperplasia, a one‐third PHx largely triggers hepatic hypertrophy.^[^ ^30^ ^]^ To date, the signaling pathways that regulate the specific percentages of hypertrophy versus hyperplasia remain largely unknown.^[^ ^23^ ^]^ Finally, liver regeneration is terminated when the liver has regained its original liver‐to‐body weight ratio.^[^ ^22^ ^]^ It was suggested that the dilation of liver sinusoids is also terminated, once the hepatic vascular bed is fully restored.^[^ ^20^ ^]^

### Hemodynamic changes in liver regeneration

In a “healthy” liver, homeostatic conditions dominate, in that both blood pressure and blood flow remain relatively constant, even though fluctuations occur during the day.^[^ ^31^ ^]^ In contrast, profound changes occur in blood flow and pressure after liver resection or liver transplantation. In mice and rats, it has been shown that the liver sinusoids dilate after PHx,^[^ ^20^ ^]^ and the diameter of sinusoids has been shown to increase by up to 0.7 µm in rats,^[^ ^32^ ^]^ which is due to the increased pressure in the portal vein. In mice the portal pressure before two‐thirds PHx is 5 mmHg and after the surgery around 8 mmHg.^[^ ^33^ ^]^ Notably, vasodilation of liver sinusoids normalizes in rats with PHx compared to sham‐operated animals after about 10 days,^[^ ^34^ ^]^ a time at which liver regeneration and restoration of the hepatic vascular beds are largely completed after PHx.^[^ ^22^ ^]^ While sinusoidal dilatation is significantly increased up to 3 days after PHx, blood flow velocity (which approximately triples right after PHx in rats) gradually decreases already 12 h after PHx.^[^ ^35^ ^]^

In humans, too much blood flow relative to the mass of the liver can lead to liver damage. This is the case when only part of a liver has been transplanted so that the liver graft is too small for the recipient. A so‐called “small‐for‐size syndrome” (SFSS) develops, in which the small liver mass cannot provide the organism with sufficient liver function and the vascular bed is too small to physiologically adapt to the blood flow of the host.^[^ ^36^ ^]^ The SFSS can lead to life‐threatening vascular complications, such as variceal bleeding and ascites.^[^ ^37^ ^]^ However, down to a certain size of the liver graft, a positive correlation exists between graft perfusion in the early postoperative period and hepatic regeneration after living donor liver transplantation.^[^ ^38^ ^]^ Overall, these reports suggest that intra‐organ blood flow plays an essential role in the growth and survival of liver tissue.

### Angiocrine signals

To date, only a few angiocrine signals with regeneration‐ or growth‐promoting effects have been identified in the liver (Table 1).^[^ ^5^, ^24^, ^39^ ^]^ One of the best‐known angiocrine signals for liver regeneration is hepatocyte growth factor (HGF).^[^ ^40^, ^41^ ^]^ In order to investigate the specific role of endothelial‐derived HGF, its expression was genetically switched off in LSECs from early fetal development onward.^[^ ^42^ ^]^ In these animals, liver regeneration was delayed for 3 days after PHx and associated with reduced hepatocyte proliferation 2 days after the surgery,^[^ ^42^ ^]^ indicating that HGF in LSECs determines the speed (rather than extent) of liver regeneration. Supporting the role of vasodilation‐induced circumferential EC stretching in organ regeneration, higher levels of HGF were detected in the secretome of mechanically stretched (compared to unstretched) hepatic ECs, indicating that LSECs release HGF in response to mechanical stimulation.^[^ ^20^ ^]^

In addition to their regenerative‐ and growth‐promoting effects, angiocrine signals also play a role in hepatic zonation. While HGF does not reveal a substantial zonation neither on mRNA nor protein level,^[^ ^43^ ^]^ hepatic zonation is regulated by angiocrine Wnts, which can also be mechanically induced.^[^ ^44^, ^45^, ^46^ ^]^ Wnts are generally important for the regulation of cell growth and maintenance of liver function,^[^ ^47^ ^]^ and in particular the angiocrine signals Wnt2 and Wnt9b play a central role in liver regeneration and are essential for hepatic zonation, as studies using an EC‐specific knockout of these Wnts have shown.^[^ ^48^, ^49^ ^]^ In addition, EC‐specific deficiency of GATA4 leads to the downregulation of Wnt2, resulting in impaired metabolic liver zonation and liver regeneration.^[^ ^50^ ^]^ Further, deficiency of ALK1 in mice was recently shown to result in a loss of Wnt2 and Wnt9b in LSECs, leading to disturbed metabolic liver zonation.^[^ ^51^ ^]^

Whereas short‐term mechanical stimulation promotes liver growth due to the release of angiocrine signals, permanent mechanical stimulation can alter release of angiocrine signals, contributing to impaired liver regeneration and health.^[^ ^20^, ^37^ ^]^ For example, in congestive hepatopathy, pathological mechanical stretch of LSECs occurs because of right ventricular heart failure and subsequent persistent sinusoidal dilation. The latter leads to Notch‐dependent secretion of the neutrophil chemoattractant C‐X‐C motif chemokine ligand 1 (CXCL1), which promotes microthrombus formation and portal hypertension.^[^ ^52^ ^]^ Endothelial Notch1 activation (or inducible EC‐specific expression of the Notch1 intracellular domain or NIC) in mice has been shown to lower the release of angiocrine signals, such as Wnt2, Wnt9b and HGF.^[^ ^53^ ^]^ In addition, Notch1 activation enlarges the area of liver sinusoids and increases the number of apoptotic hepatocytes compared to controls. It also enhances carbon tetrachloride (CCl_4_)‐induced liver fibrosis in mice.^[^ ^53^ ^]^ The strength, duration and mode of mechanical stimulation of ECs by blood flow or blood pressure can therefore be a stimulus that determines the pattern of angiocrine signals (as well as degree and length of exposure to these signals), which can either promote liver regeneration or culminate in liver failure. The notion that there are angiocrine signals that favor liver regeneration and angiocrine signals that interfere with it was shown by Ding et al.^[^ ^40^ ^]^ In acute liver injury induced by a single injection of CCl_4_ or by acetaminophen, upregulation of CXCR7 in LSECs leads to the release of angiocrine signals (HGF and Wnt2) that promote liver regeneration.^[^ ^40^, ^54^ ^]^ In contrast, repeated injections of CCl_4_ upregulate CXCR4 in LSECs, so that angiocrine signals (for example transforming growth factor β or TGFβ, bone morphogenetic protein 2 or BMP2 and platelet‐derived growth factor C or PDGF‐C) are released that favor liver fibrosis.^[^ ^54^ ^]^

### MYDGF—a novel angiocrine signal in liver regeneration

PHx leads to increased hepatic perfusion in mice, ultimately promoting liver growth through vasodilation and mechanically‐triggered release of signals required for liver regeneration (Table 1), for example, HGF, IL‐6, and TNF‐α.^[^ ^20^ ^]^ Recently, we screened for novel mechanically‐induced angiocrine signals by analyzing proteins released by LSECs upon mechanical stretching (thereby mimicking the mechanical effects of vasodilation on LSECs) and identified myeloid‐derived growth factor (MYDGF) as a novel angiocrine signal.^[^ ^55^ ^]^

MYDGF has previously been shown to play an important role in cardiac repair after heart injury,^[^ ^56^, ^57^, ^58^ ^]^ but its role in liver regeneration was unknown. We therefore analyzed *Mydgf* deficient mice as well as mice with liver‐specific *Mydgf* overexpression. While these mice showed no liver defects under baseline conditions^[^ ^56^ ^]^ (our unpublished data), liver regeneration in *Mydgf* deficient mice seemed to be impaired due to massively reduced hepatocyte proliferation after performing a two‐thirds PHx. This was shown by analyzing liver tissue early after PHx.^[^ ^55^ ^]^ In contrast, mice with liver‐specific *Mydgf* overexpression showed accelerated liver regeneration with an increased proliferation rate of hepatocytes early after PHx compared to control mice.^[^ ^55^ ^]^ Since MYDGF did not affect hepatocyte proliferation under baseline conditions, MYDGF does not seem to be sufficient to induce proliferation but appears to require an additional signal (for exampl an injury‐based signal or another angiocrine signal) to do so.

**FIGURE 2:**
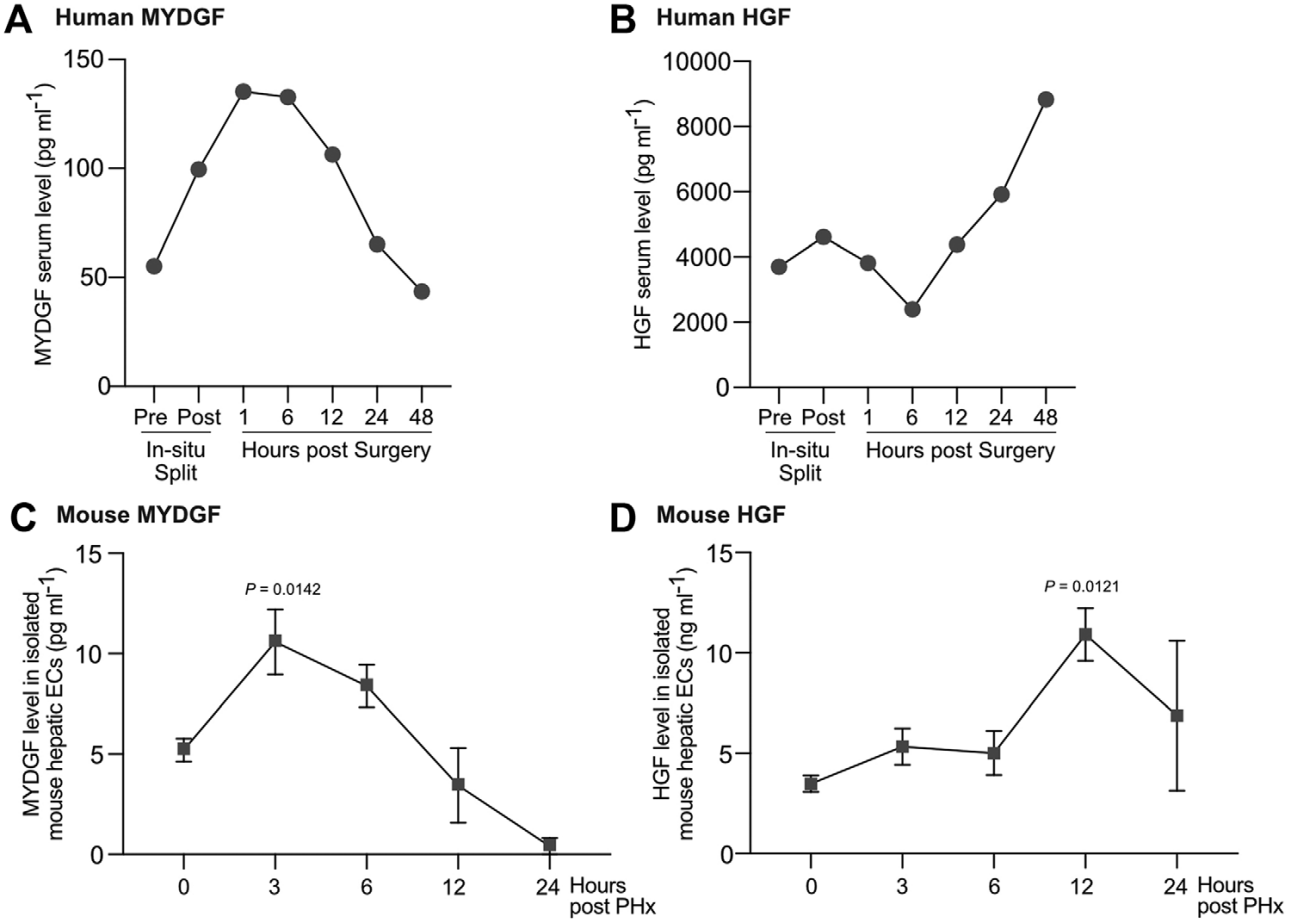
MYDGF versus HGF concentrations after liver surgery in humans and mice. Analysis of serum MYDGF (A) or HGF (B) levels after stage 1 of in‐situ split liver surgery, *n* = 1 male patient. Analysis of MYDGF (C) or HGF (D) levels in mouse hepatic ECs isolated from the remaining right liver lobe at different time points after PHx. *N* = 5 (0 and 3 h), *n* = 4 (6 and 12 h) and *n* = 3 mice (24 h). Data are presented as mean ± SEM. *p*‐values were calculated using one‐way ANOVA followed by Dunnett's post hoc test (C, D). Part of this Figure was published in ^[^ ^55^ ^]^, which is licensed under the Creative Commons Attribution 4.0 International License. EC, endothelial cells; HGF, hepatocyte growth factor; MYDGF, myeloid‐derived growth factor; PHx, partial hepatectomy.

Recombinant MYDGF was found to promote proliferation of primary human hepatocytes via mitogen‐activated protein kinase (MAPK) and signal transducer and activator of transcription 3 (STAT3) signaling.^[^ ^55^, ^59^, ^60^ ^]^ However, the receptor (or direct target) of MYDGF has not yet been identified. Along with its proliferation‐promoting effect on hepatocytes, an increase of MYDGF concentrations can be observed in the plasma of human individuals shortly after liver resection, as well as in mouse liver and isolated hepatic ECs early after PHx.^[^ ^55^ ^]^ Strikingly, while blood concentrations of HGF peak 24 h after liver resection in humans^[^ ^61^, ^62^, ^63^ ^]^ and 12 h after PHx in mice,^[^ ^42^ ^]^ MYDGF concentrations already increase within the first hour after liver resection in humans and 3 h after PHx in mice (Figure 2), indicating that MYDGF plays a role in the initiation or priming phase of liver regeneration. However, MYDGF blood concentrations are substantially lower than the respective HGF concentrations.^[^ ^55^ ^]^ This does not restrict its role, in particular when MYDGF is released locally in the liver and binds to a yet‐to‐be‐identified, high‐affinity receptor. In the future, identification of the latter could uncover a promising signaling pathway with therapeutic impact on liver regeneration and heart repair.

In the following sections, we will address the question of whether an enhanced blood flow also plays a role during the regeneration of other inner organs, in particular lungs and kidneys.

## COMPENSATORY LUNG GROWTH IS INITIATED BY HEMODYNAMIC CHANGES AFTER PNEUMONECTOMY

The lung is the main organ for gas exchange, that is, oxygen uptake and carbon dioxide disposal, and is the central part of the pulmonary circulation; blood is pumped directly from the right ventricle to the lung and, therefore, the pulmonary vascular system cannot be bypassed. Hemodynamic changes have been observed after partial lung resection (pneumonectomy, PNX), which is often performed in human individuals with lung disease or cancer and can be experimentally mimicked in multiple animal models, including mice.^[^ ^2^, ^64^, ^65^ ^]^ In particular in children and adolescents, PNX induces lung growth, resulting in an increased residual lung volume that finally compensates for the resected lung tissue (Figure 1B).

In contrast to the rapid regeneration of the liver, lung regrowth takes several months to years in humans,^[^ ^66^ ^]^ while in mice the lung capacity is completely restored within weeks.^[^ ^67^ ^]^ However, compensatory lung growth declines with age, similar to the impairment of liver regeneration observed in aged mice.^[^ ^68^, ^69^ ^]^ The mechanisms underlying compensatory lung growth are not fully understood, but mechanical forces are most likely an essential initial signal. This is because blood flow is forced through the remaining part of the lung and, therefore, stretching of ECs (besides mechanical stimulation of lung epithelial cells by other means) can be assumed.^[^ ^2^, ^70^, ^71^, ^72^ ^]^ Indeed, a substantial increase in pulmonary artery (PA) pressure (1.8‐fold increase in right PA pressure after left unilateral PNX in mice) occurs when the entire cardiac output from the right ventricle has to flow through the remaining lung tissue.^[^ ^71^ ^]^ Consistently, pulmonary vascular resistance also increases after PNX,^[^ ^73^ ^]^ likely due to the reduced pulmonary vascular bed.

At the cellular level, PNX leads to the growth of existing alveoli and formation of new alveolar units (neo‐alveolarization) followed by angiogenesis.^[^ ^74^, ^75^, ^76^, ^77^ ^]^ More specifically, in mice, the number of alveoli almost reaches baseline levels 3 weeks after PNX, with 74% of new alveoli formed by hyperplasia within the first 6 days after PNX.^[^ ^74^ ^]^ Specifically, the number of alveoli increases through the proliferation of alveolar type 2 (AT2) cells followed by an AT2‐into‐AT1 (alveolar type 1) cell differentiation.^[^ ^71^, ^78^, ^79^ ^]^ Formation of new alveolar septa (including capillaries and mesenchymal cells) takes place from pre‐existing septa of the remaining lung, similar to developmental alveolarization.^[^ ^76^ ^]^ AT1 cells constitute around 95% of the alveolar surface and are important for gas exchange, while AT2 cells produce pulmonary surfactant and act as alveolar progenitors for lung repair after injury.^[^ ^79^, ^80^ ^]^ Normal alveolar cell turnover is low,^[^ ^81^ ^]^ but increases after lung injury, with a peak in AT2 proliferation observed 5 days after left unilateral PNX in mice.^[^ ^82^ ^]^ At this time point, nuclear expression of Yes‐associated protein 1 (YAP1) is increased in AT2 cells.^[^ ^82^ ^]^ YAP1 is activated by mechanical stimuli, including shear stress or mechanical tension ^[^ ^82^, ^83^ ^]^, and it is required for the AT2‐to‐AT1 cell differentiation after lung injury.^[^ ^82^, ^84^, ^85^ ^]^ When *Yap* is deleted in AT2 cells, the proliferation of AT2 cells after PNX is reduced by almost 50%, 5 days after PNX in mice, whereas AT1‐specific deletion of *Yap* has no effect on AT2 proliferation at this time point.^[^ ^82^ ^]^ However, YAP1 also plays a role in ECs after PNX, since an EC‐specific knockdown of *Yap1* was shown to reduce compensatory lung growth, which is reflected by impaired vascular and alveolar morphogenesis after left unilateral PNX.^[^ ^86^ ^]^ The results therefore point to mechanically induced YAP1‐dependent angiocrine signals from pulmonary capillary ECs.^[^ ^87^ ^]^

Since increased mechanical tension occurs in the remaining lung after PNX, the mechanistic concepts reported for liver regeneration may be (at least in part) applicable to the lung. Of note, left unilateral PNX was shown to activate VEGFR2 and fibroblast growth factor receptor‐1 (FGFR1) on pulmonary capillary ECs, stimulating the production of the matrix metalloproteinase 14 (MMP14).^[^ ^88^ ^]^ The latter unmasks cryptic epidermal growth factor receptor (EGFR) ligands (shedding of heparin‐binding epidermal growth factor or HB‐EGF and cleavage of the laminin‐5 gamma 2 chain), which in turn stimulate proliferation of AT2 cells.^[^ ^88^ ^]^ In line with this scenario, EC‐specific deletion of *Vegfr2* plus *Fgfr1* inhibited the production of MMP14, leading to impaired alveolarization and compensatory lung growth, which could be restored by intravascular transplantation of MMP14^+^ pulmonary capillary ECs.^[^ ^88^ ^]^ Likewise, EC‐specific deletion of *Mmp14* in mice resulted in reduced AT2 cell proliferation after left unilateral PNX, while EC proliferation remained unchanged.^[^ ^89^ ^]^ Besides MMP14, stromal cell‐derived factor 1 (SDF‐1; also known as CXCL12) plays a role in compensatory lung growth. SDF‐1 is released by activated platelets, which activate SDF‐1 receptors CXCR4 and CXCR7 on pulmonary capillary ECs.^[^ ^89^ ^]^ The binding of SDF‐1 to its receptors leads to the deployment of MMP14 in pulmonary capillary ECs, resulting in the release of HB‐EGF, which promotes AT2 cell proliferation.^[^ ^89^ ^]^ Consistently, mice lacking platelets or harboring a platelet‐specific deletion of *Sdf‐1* showed reduced alveolarization after PNX, an effect that could be rescued by an intravascular infusion of (SDF‐1 positive) platelets.^[^ ^89^ ^]^

In addition to AT2 cell proliferation, also pulmonary angiogenesis has been shown to take place after PNX, and this angiogenesis is partly reminiscent of blood vessel formation during embryonic lung development.^[^ ^68^, ^71^, ^76^, ^86^, ^88^, ^90^ ^]^ For example, sprouting and non‐sprouting (intussusceptive) angiogenesis occur in the lungs of mice 3–6 days after PNX.^[^ ^76^, ^91^ ^]^ Furthermore, increased plasma VEGF‐A levels could be observed in mice after left unilateral PNX.^[^ ^92^ ^]^ In line with a role of VEGF‐A in lung regeneration, exogenous intraperitoneal injection of VEGF‐A164 (containing a heparin‐binding domain, HB‐D) led to faster compensatory lung growth after left unilateral PNX in mice,^[^ ^93^, ^94^ ^]^ whereas intraperitoneal injection of neutralizing antibodies against VEGF‐A suppressed compensatory lung growth after PNX.^[^ ^92^ ^]^ Intraperitoneal injection of VEGF‐A120 (i.e., VEGF‐A without HB‐D) did not lead to increased pulmonary cell proliferation in the remaining mouse lung,^[^ ^94^ ^]^ suggesting that compensatory lung growth requires specific VEGF‐A variants and angiogenesis.

In conclusion, the interplay between mechanical forces, angiocrine signaling, epithelial progenitor cell proliferation, and angiogenesis support compensatory lung growth after PNX.

## COMPENSATORY RENAL GROWTH IS ASSOCIATED WITH HEMODYNAMIC CHANGES AFTER NEPHRECTOMY

The kidneys represent the largest blood‐filtering system of the mammalian body, receiving approximately 20% (or around 1 L blood) of cardiac output.^[^ ^95^ ^]^ In mice and humans, an entire kidney (radical nephrectomy) or a large part thereof can be removed. The resection is associated with the growth of the remaining kidney (which is called compensatory renal hypertrophy or CRH, even though it involves both hypertrophy and hyperplasia) (Figure 1C).^[^ ^3^, ^96^ ^]^. During CRH, the size and weight of the remaining kidney increases. In mice, the weight of the remaining kidney (normalized to body weight) increases 24 h after nephrectomy and reaches a plateau after 3 days, with the weight of the kidney increasing by approximately 25% compared to sham‐operated mice.^[^ ^96^ ^]^ Notably, growth of the remaining kidney or CRH involves an angiogenic response,^[^ ^97^, ^98^ ^]^ and the growth of the existing nephrons is characterized by hypertrophy and hyperplasia.^[^ ^96^ ^]^ During the early stages of CRH, mouse proximal tubule cells show hypertrophy, while the cortical collecting ducts exhibit hyperplasia.^[^ ^96^ ^]^ Kikuchi et al. showed that the lipid‐activated transcription factor peroxisome proliferator‐activated receptor alpha (PPARα) plays an important role in the hypertrophy of proximal tubule cells. This was demonstrated by *PPARα* knockout mice, which had a smaller remaining kidney and a significantly reduced volume of proximal tubule cells 3 days after nephrectomy.^[^ ^96^ ^]^

The mechanisms, by which the remaining kidney senses the loss of the other kidney and triggers CRH after unilateral nephrectomy, are poorly understood. However, hemodynamic changes (for example increased renal blood flow or RBF) and an enhanced workload (for example increased glomerular filtration rate or GFR) in the remaining kidney, as well as the reduced release of kidney‐specific circulating factors (for example renin), may play a role.^[^ ^3^ ^]^ More specifically, an increase in RBF and single‐nephron GFR has been observed in the remaining kidney after unilateral nephrectomy in animals and humans.^[^ ^98^, ^99^, ^100^, ^101^ ^]^ The mechanisms that lead to increased RBF after nephrectomy are not fully understood, but the renin‐angiotensin system (RAS) may play a critical role. Since the kidney produces renin, the levels of the latter decrease after nephrectomy due to the removal of one kidney.^[^ ^102^ ^]^ Shimada et al. showed that the immediate increase in RBF after unilateral right nephrectomy in rats is blocked by the angiotensin 2 type 1 receptor blocker losartan and the renin inhibitor aliskiren.^[^ ^98^ ^]^ However, chronically increased RBF and hypertrophy of the remaining kidney are not prevented by RAS inhibition,^[^ ^98^ ^]^ suggesting that RAS is only necessary for the immediate increase in RBF after nephrectomy.

The importance of hemodynamic changes for CRH has been shown in eNOS‐deficient mice, which displayed a reduced hemodynamic response after nephrectomy and impaired CRH after unilateral nephrectomy^[^ ^103^ ^]^, quite similar to what has been observed in the regenerating liver.^[^ ^104^ ^]^ Thus, it is conceivable that hemodynamic changes in the remaining kidney after nephrectomy lead to NO release, vasodilation, and concomitant mechanical stretching of microvascular ECs, similar to the observations made in the liver,^[^ ^20^ ^]^ resulting in the release of angiocrine signals (Table 1). The latter could promote growth of renal tubular epithelial cells after nephrectomy. In the study by Tasnim and Zink, it was shown that there is indeed a crosstalk between ECs and renal tubular epithelial cells since co‐culture of both cell types leads to the release of angiocrine growth factors, such as HGF and TGFβ1.^[^ ^105^ ^]^ Notably, exogenous HGF treatment increases DNA synthesis in tubular epithelial cells after unilateral nephrectomy in mice,^[^ ^106^ ^]^ and TGFβ has been described to induce hypertrophy in rat renal tubular cells.^[^ ^107^ ^]^ In a recent study, Kikuchi et al. used a multiomics approach to identify additional signaling mechanisms in the residual kidney after nephrectomy.^[^ ^96^ ^]^ Their proteomics data revealed an increase in angiocrine signals, such as MYDGF and insulin‐like growth factor 1 (IGF1), in the remaining kidney of mice 24 h after unilateral nephrectomy.^[^ ^96^ ^]^ Notably, the stretch‐induced angiocrine signal MYDGF has been described in the kidney to protect from several glomerular kidney diseases.^[^ ^108^, ^109^ ^]^ While deletion of *Mydgf* in mice had no effect on kidney size,^[^ ^56^ ^]^ its ablation led to increased podocyte injury, proteinuria, and glomerular hypertrophy in mouse models with diabetic kidney disease (DKD) or focal segmental glomerulosclerosis mouse models. Further, recombinant MYDGF prevented podocyte injury in vitro and in vivo.^[^ ^108^, ^109^ ^]^ Thus, MYDGF represents an angiocrine signal that is possibly secreted by renal ECs after nephrectomy to promote kidney health and growth after nephrectomy.

In conclusion, the kidney's response to nephrectomy involves a complex interplay of hemodynamic changes and cellular responses in the parenchyma, leading to compensatory renal hypertrophy (and hyperplasia). Further research is needed to clarify the underlying mechanisms and influence of angiocrine signaling on CRH.

## COMMON ASPECTS AND DIFFERENCES IN ORGAN REGENERATION

**FIGURE 3:**
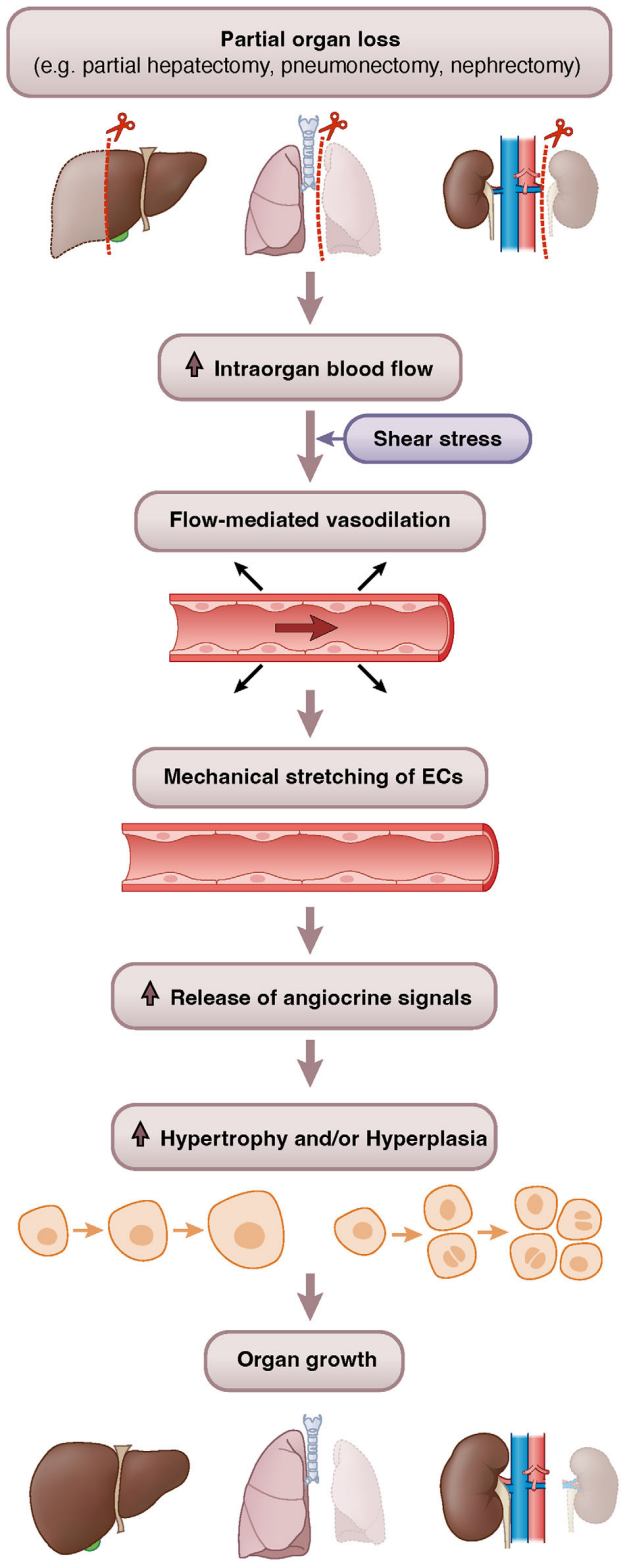
General model: Hemodynamic changes due to partial organ loss promote organ growth via mechanically‐released angiocrine signals. After partial organ loss, such as after partial hepatectomy, pneumonectomy or nephrectomy, the respective intra‐organ blood flow increases. The hemodynamic changes lead to mechanical stretching of the organ‐specific microvascular ECs, which subsequently release angiocrine signals to induce hypertrophy and/or hyperplasia of the tissue cell types, thus promoting organ growth. EC, endothelial cells.

In all three organs described in this review, partial or complete removal of the organ leads to compensatory growth of the remaining tissue. The latter is induced (at least in part) by hemodynamic changes within the remaining part of the organ (Figure 3). In the liver, intra‐organ blood flow increases after PHx due to an increase in portal blood flow relative to organ mass, while in the lung PA pressure, and the remaining kidney RBF increases. Particularly in the liver, the enhanced blood flow leads to shear stress in the sinusoidal blood vessels and flow‐mediated vasodilation (FMD).^[^ ^20^ ^]^ This stimulates mechanical stretching of hepatic ECs, which then release angiocrine signals that stimulate hepatocyte proliferation and thereby liver regeneration.^[^ ^20^, ^55^ ^]^ To our knowledge, a similar mechanism has not yet been demonstrated in detail in the lungs and kidneys. However, since hemodynamic changes also occur in these organs after PNX and nephrectomy, respectively, FMD could also lead to EC stretching and thereby secretion of mechanically‐induced, growth‐promoting angiocrine signals there. Of note, in the organs mentioned, the process of regeneration or compensatory growth is slowed down or even impaired with an advanced age.^[^ ^68^, ^69^, ^110^, ^111^ ^]^

Besides the similarities mentioned above, the three organs also exhibit differences in how they adapt to the loss of a portion of their tissue. While the liver regains its mass within 2 weeks in humans, the compensatory growth of the lung and kidney takes weeks to months.^[^ ^17^, ^66^, ^112^, ^113^ ^]^ In addition, in rodents, angiogenesis occurs within the first week in the liver and lung after PHx and PNX, respectively,^[^ ^23^, ^76^ ^]^ while it starts a week later in the kidney after radical nephrectomy,^[^ ^114^ ^]^ indicative of an extended angiocrine period in this organ system.

## FUTURE DIRECTIONS

Even though not the topic of this review, a lot work has been recently published on angiocrine signals in tumor cell growth and dissemination.^[^ ^115^, ^116^ ^]^ For example, the suppression of angiocrine CC chemokine ligand 2 (CCL2) by adrenomedullin was shown to promote tumor growth.^[^ ^117^ ^]^ Since CCL2 has principally been shown to be mechano‐responsive,^[^ ^118^ ^]^ it would be interesting to analyze whether angiocrine secretion of CCL2 could be stimulated by an increased blood flow or increased blood/interstitial pressure in tumor tissue. Further, it has recently been shown that angiocrine Wnt signaling promotes the exit of tumor cells from the blood vessels (in a process called extravasation) and causes tumor latency in the lung.^[^ ^119^ ^]^ Since Wnt/β‐catenin signaling in ECs has been shown to be mechanically regulated,^[^ ^46^ ^]^ mechanically‐induced angiocrine signals would be worth investigating for their role in both, tumor growth and tumor cell dissemination. In general, since ECs actively shape the tumor microenvironment by releasing multiple angiocrine signals, for example, Ang‐2, basic Fibroblast Growth Factor (bFGF or FGF2), CCL2, EGF, endothelin 1, IL‐6, IL‐8, TGFβ, platelet‐derived growth factor‐β (PDGFβ), intracellular adhesion molecule 1 (ICAM‐1), vascular cell adhesion molecule (VCAM), angiomodulin (IGFBP7) and Jagged 1 (JAG1),^[^ ^115^, ^120^, ^121^, ^122^ ^]^ further research on their potential mechanical regulation is warranted.

## CONCLUSION

Partial organ loss, such as after PHx, PNX, or nephrectomy, alters intra‐organ blood flow (Figure 3). Hemodynamic forces, including shear stress that is followed by FMD cause microvascular ECs to be mechanically stimulated after partial organ loss. The latter stimuli trigger the release of angiocrine signals to promote organ hypertrophy and/or hyperplasia (Figure 3).^[^ ^20^, ^28^, ^55^ ^]^ Even though mechanical aspects also play a key role in cancer biology, direct links between mechanical stimuli, angiocrine signals, and cancer cell behavior are still missing. A great perspective for mechanically‐induced angiocrine signals currently is their clinical application to accelerate organ growth and regeneration after organ injury or partial organ removal.

## AUTHOR CONTRIBUTIONS

Paula Follert, Linda Große‐Segerath and Eckhard Lammert wrote the manuscript and drafted the figures. Paula Follert prepared the graphical abstract with the help from Eckhard Lammert. All authors read and contributed to the manuscript.

## CONFLICT OF INTEREST STATEMENT

The authors declare no conflict of interest.

## Data Availability

The data that support the findings of this study are available from the corresponding author upon reasonable request.
